# Parents' Perception on Post-tonsillectomy Hemorrhage: A Local Survey

**DOI:** 10.7759/cureus.62907

**Published:** 2024-06-22

**Authors:** Hassan F Alkhars, Ossama M Zakaria, Hussam Alkhars

**Affiliations:** 1 Otolaryngology, King Fahad General Hospital, Hofuf, SAU; 2 Medicine and Surgery, King Faisal University, Hofuf, SAU

**Keywords:** hemorrhage, saudi arabia, knowledge, health care, parent's perception, bleeding, complications, tonsillectomy

## Abstract

Introduction: Tonsillectomy (often combined with adenoidectomy) is one of the oldest and most common surgical procedures performed in otolaryngology. Post-operative complications following tonsillectomy are generally rare and include post-tonsillectomy hemorrhage, dehydration, velopharyngeal insufficiency, and others. Parents play a crucial role in the care and recovery of their children after tonsillectomy. Their perception and understanding of post-tonsillectomy hemorrhage are essential in managing and addressing this potential complication.

Aim: The purpose of this study is to assess parents' perception of post-tonsillectomy hemorrhage and factors that would lead to its development.

Methods: A descriptive cross-sectional study was conducted targeting parents of children who had undergone surgical tonsillectomy. Data were collected using a pre-structured online questionnaire, biographical data, tonsillectomy data, child medical and drug history, healthcare staff role, and post-surgical complications and bleeding.

Results: A total of 847 parents completed the study questionnaire, and 431 (50.9%) were fathers. As for education, 164 (19.4%) had a university level of education, and 279 (32.9%) had a post-graduate degree. As for child gender, 445 (52.5%) were males, 232 (27.4%) had undergone the surgery in the first five years of their age, 208 (24.6%) at the age of 6-10 years, and 221 (26.1%) undergone the surgery at the age of 16-18 years. The most reported post-surgical complications included headaches and nausea (52.4%), swelling of the roof of the mouth (51.8%), and infection (48.9%). Primary or secondary post-tonsillectomy hemorrhage was reported among 47 (5.5%) of the children, which was during surgery among 12 (25.5%), within 24 hours after surgery among 14 (29.8%), and after 24 hours of surgery among 21 (44.7%).

Conclusion: The current study revealed a high rate of tonsillectomy-associated bleeding with a shortage regarding the role of healthcare staff in child pre-surgical assessment and also in parents' education regarding expected complications.

## Introduction

Tonsillectomy and adenoidectomy are frequently performed surgeries worldwide. However, up until the year 1984, their effectiveness was not heavily studied. When comparing pediatrics and adult research on these surgeries, pediatrics has more extensive and deep studies in this matter. Looking at the current guidelines in pediatrics, surgery has been recommended for patients with pediatric obstructive sleep apnea syndrome and recurrent tonsillitis [1]. Tonsillectomies are typically known to be safe procedures with minimum complication rates. However, post-tonsillectomy hemorrhage remains a major concern following the procedure. Post-tonsillectomy hemorrhage is divided into two types. The first type is bleeding that occurs within 24 hours after surgery, which is called primary post-tonsillectomy hemorrhage, while the other type is bleeding that occurs after 24 hours of the surgery, which is called secondary post-tonsillectomy hemorrhage. In severe or uncontrolled cases of post-tonsillectomy hemorrhage, additional surgery may be necessary to manage the hemorrhage [2,3]. Primary post-tonsillectomy hemorrhage has been associated with various factors, including the surgical technique used, insufficient contraction of the blood vessels in the tonsillar area, and impaired blood clotting. On the other hand, secondary post-tonsillectomy hemorrhage is associated with factors such as gender, age, the use of non-steroidal anti-inflammatory drugs (NSAIDs), inadequate oral intake following the surgery, and seasonal variations [2,4,5].

One of the studies previously done found that patients who were taking anti-inflammatories (ibuprofen) were at higher risk of developing post-tonsillectomy hemorrhage than patients who were not. Moreover, as individuals get older, the risk of developing post-tonsillectomy hemorrhage increases following the procedure. Similarly, another study showed that the skill level and the experience of the surgeon also played a major role in the incidence of post-tonsillectomy hemorrhage [6]. Trainee-level surgeons had a higher risk of post-tonsillectomy bleeding compared to senior consultants. When comparing different tonsillectomy procedure techniques, a study showed that patients undergoing bipolar diathermy had a greater risk of developing post-tonsillectomy hemorrhage compared to those undergoing the cold dissection technique [7].

In Saudi Arabia, childhood obesity is more prevalent than in other countries. It is important to investigate whether childhood obesity has increased the incidents of post-tonsillectomy hemorrhage in our population [8,9].

Parents play an important role in the postoperative care of their child following a tonsillectomy. Their perception and understanding of the procedure are essential elements in addressing and managing the various potential complications, especially post-tonsillectomy hemorrhage.

The aim of this research is to investigate parents' perception of post-tonsillectomy hemorrhage following their child’s tonsillectomy. It will explore the knowledge, attitudes, and experiences of parents regarding post-tonsillectomy hemorrhage. By understanding their perception, healthcare providers would be able to develop a strategy to address parents' concerns, improve patient satisfaction, and improve overall pre- and post-operative care.

## Materials and methods

We carried out a cross-sectional study from January to April 2024 in Saudi Arabia. We included children up to 18 years of age who had tonsillectomy. With a pre-determined level, the sample size was calculated to be 385. We used non-probability convenience sampling to recruit parents who met the inclusion criteria. We distributed an online questionnaire created in Google Forms through social media channels to parents selected via simple random sampling. The questionnaire was written in Arabic and divided into five sections. The first section focused on demographics. The second section signs and symptoms that were present in the patient. The third section included pre-operative care that was provided. The fourth section included pre-operative conditions and co-morbidities that the patient had. The fifth section included post-operative complications and bleeding. Subscale scores were computed by taking the mean value for items associated with each subscale. The total score referred to as acceptance was computed by calculating the mean across all items.

The questionnaire was designed in Arabic language, and its linguistic clarity was verified by language experts. The Lawshe method was employed to assess the content validity of the questionnaire. Five experts were consulted to provide their opinions on each item in the questionnaire, and the content validity ratio was calculated accordingly. Questions with a content validity ratio below 0.99 were eliminated from the questionnaire. The construct validity and reliability of the questionnaire were assessed in a pilot study involving 95 participants. However, the data collected from these participants were not included in the final analysis and dissemination of the questionnaire results.

The data were collected, reviewed, and then fed to Statistical Product and Service Solutions (SPSS, version 26; IBM Corp., Armonk, NY). All statistical methods used were two-tailed with an alpha level of 0.05 considering significance if the P value is less than or equal to 0.05. Descriptive analysis was done by prescribing frequency distribution and percentage for categorical study variables, including demographic data, comorbidities, surgery data, medication, pre-operative care, and post-operative complications and bleeding. Child clinical presentations were graphed. Crosstabulation was done to assess factors associated with bleeding history among children undergone tonsillectomy surgery using Pearson's chi-square test and exact probability test for small frequency distributions.

## Results

A total of 847 parents completed the study questionnaire, and 431 (50.9%) were fathers. As for education, 164 (19.4%) had a university level of education, and 279 (32.9%) had post-graduate degrees. A total of 266 (31.4%) were not working, and 308 (36.4%) were employees. Monthly income less than 5,000 SR was reported among 228 (26.9%), and 210 (24.8%) had monthly income exceeding 20,000 SR. As for child gender, 445 (52.5%) were males, 232 (27.4%) had undergone surgery at the first five years of their age, 208 (24.6%) at the age of 6-10 years, and 221 (26.1%) undergone surgery at the age of 16-18 years. Exactly 425 (50.2%) of the children underwent the surgery in the summer (Table 1).

**Table 1:** Personal characteristics of the study parents and surgery data (n=847) The data were represented as (N) and (%) for participants in each question.

Personal data	No	%
Responder		
Mother	431	50.9%
Father	416	49.1%
Educational level		
Illiterate	130	15.3%
Basic education	130	15.3%
Diploma	144	17.0%
University education	164	19.4%
Post-graduate	279	32.9%
Work		
Not working	266	31.4%
Student	273	32.2%
Employee	308	36.4%
Monthly income		
< 5000 SR	228	26.9%
5000-10,000 SR	190	22.4%
10,000-20,000 SR	219	25.9%
> 20,000 SR	210	24.8%
Child gender		
Male	445	52.5%
Female	402	47.5%
Child age at the time of surgery		
1-5 years	232	27.4%
6-10 years	208	24.6%
11-15 years	186	22.0%
16-18 years	221	26.1%
Season at the time of surgery		
Winter	422	49.8%
Summer	425	50.2%

**Figure 1:**
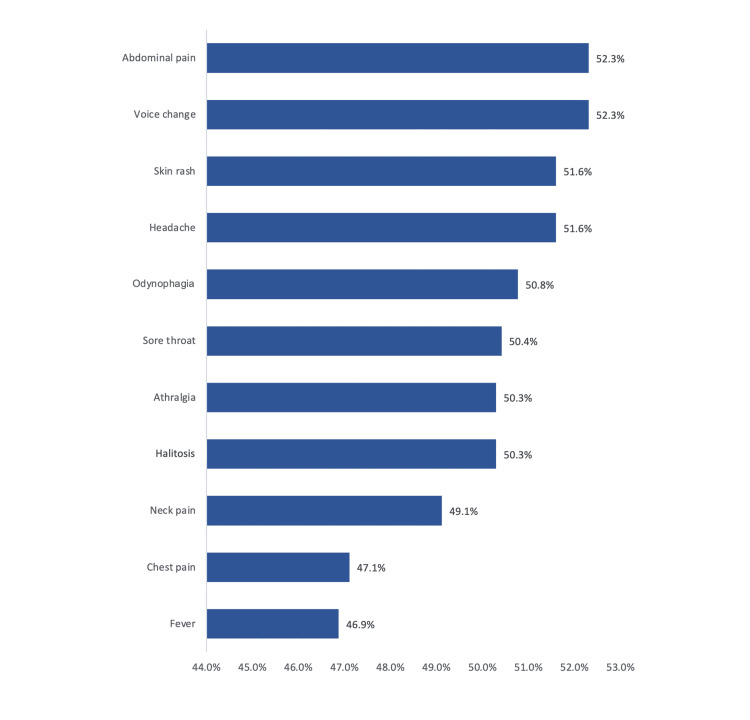
Clinical symptoms among study children who underwent tonsillectomy surgery The data were represented as (%) for participants in each question.

Figure 1 presents the clinical symptoms among study children who underwent tonsillectomy surgery. The most reported symptoms included abdominal pain (52.3%), voice change (52.3%), skin rash (51.6%), headache (51.6%), and odynophagia (50.8%). The least reported were neck pain (49.1%), chest pain (47.1%), and fever (46.9%).

With regard to the preoperative healthcare among study children (Table 2), 51.2% of the children did laboratory tests before the operation by the treating medical staff, and 49.1% showed anemia. As for explanations about expected complications, 52.2% were informed about post-operative bleeding, 50.9% about headache and nausea, 49.9% about intra-operative bleeding, 49.4% about swelling of the roof of the mouth, and 48.9% about infection. A total of 396 (46.8%) of the parents said that medical staff told them how to deal with the complications described above if they occur.

**Table 2:** Preoperative healthcare among study children The data were represented as (N) and (%) for participants in each question.

Variable	No	%
Complete laboratory tests were performed before the operation by the treating medical staff?		
Yes	434	51.2%
No	413	48.8%
Did any of the tests show the presence of anemia?		
Yes	416	49.1%
No	431	50.9%
Did the health care staff mention the expected complications?		
Headache and nausea	431	50.9%
Swelling of the roof of the mouth	418	49.4%
Intra-operative bleeding	423	49.9%
Post-operative bleeding	442	52.2%
Infection	414	48.9%
Did the medical staff tell you how to deal with the complications described above if they occur?		
Yes	396	46.8%
No	451	53.2%

Table 3 presents the preoperative health condition of the study children and medical staff role. Considering child comorbidities, 436 (51.5%) had morbid obesity, 437 (51.6%) had chronic tonsillitis, and 185 (21.8%) had rheumatic fever. Hemophilia was detected among 95 (11.2%) children. A total of 781 (92.2%) of the parents reported that the treating medical staff asked about the comorbidities described above before the operation. Exactly 72 (8.5%) of the children were on anti-inflammatories (ibuprofen) before surgery, 311 (36.7%) were asked about this treatment by the medical staff, and 49 (68.1%) of them were directed to stop treatment by the treating medical staff. A total of 31 (63.3%) stopped this medication one to two weeks before the surgery, and 11 (22.4%) stopped one to six days before. A total of 268 (31.6%) of the children were on anticoagulants or aspirin, 782 (92.3%) were asked about it by the medical staff, and 233 (86.9%) were directed to stop it. Aspirin was given for rheumatic fever among 109 (40.7%) of the users. Exactly 392 (46.3%) committed the treating medical staff instruction before and after the surgery. 

**Table 3:** Preoperative health condition of the study children and medical staff role The data were represented as (N) and (%) for participants in each question.

Variable		No	%
Does your child have any of the following comorbidities?	Bleeding disorders (hemophilia)	95	11.2%
	Morbid obesity	436	51.5%
	DM	95	11.2%
	Chronic tonsillitis	437	51.6%
	Rheumatic fever	185	21.8%
Did the treating medical staff ask about the comorbidities​​​​​​​ described above before the operation?	Yes	781	92.2%
	No	66	7.8%
If not, which medical health condition was not mentioned?	Bleeding disorders (hemophilia)	11	16.7%
	Chronic tonsillitis	12	18.2%
	DM	22	33.3%
	Morbid obesity	21	31.8%
Is your child under anti-inflammatories (ibuprofen)?	Yes	72	8.5%
	No	775	91.5%
Did the treating medical staff ask about the treatment described above before the operation?	Yes	311	36.7%
	No	536	63.3%
If your child is under anti-inflammatories (ibuprofen) and the treating medical staff asked about it before the operation, were you directed to stop the treatment by the treating medical staff? (Answer no if they did not ask and your child is taking the medication)	Yes	49	68.1%
	No	23	31.9%
If yes, how long before the surgery were you instructed to stop the medication?	1-6 days	11	22.4%
	1-2 weeks	31	63.3%
	3-4 weeks	7	14.3%
Is your child under anticoagulants​​​​​​​ or aspirin?	Yes	268	31.6%
	No	579	68.4%
Did the treating medical staff ask about the treatment described above before the operation?	Yes	782	92.3%
	No	65	7.7%
If your child is under anticoagulants​​​​​​​ or aspirin and the treating medical staff asked about it before the operation, were you directed to stop the treatment by the treating medical staff? (Answer no if they did not ask and your child is taking the medication)	Yes	233	86.9%
	No	35	13.1%
If yes, how long before the surgery were you instructed to stop the medication?	1-6 days	33	14.2%
	1-2 weeks	184	78.7%
	3-4 weeks	16	6.7%
if your child is under aspirin, is it used for rheumatic fever or another condition?	Rheumatic fever	109	40.7%
	Other condition	159	59.3%
Were all recommended directions of the treating medical staff followed before and after the surgery?	Yes	392	46.3%
	No	455	53.7%

Table 4 presents the data on post-surgical complications and bleeding. The most reported post-surgical complications included headaches and nausea (444, 52.4%), swelling of the roof of the mouth (439, 51.8%), and infection (414, 48.9%). Bleeding was reported among 47 (5.5%) of the children, which was distributed as follows: during surgery (12, 25.5%), within 24 hours after surgery (14, 29.8%), and after 24 hours of surgery (14, 44.7%). Intra-operative or primary post-tonsillectomy hemorrhage was managed in the operating room (OR) among nine (34.6%), and 17 (65.4%) were treated conservatively. Additionally, nine (42.9%) of cases with secondary post-tonsillectomy hemorrhage went to the emergency department, where 12 (57.1%) stayed at home and resolved. Two (22.2%) of the cases that went to the emergency department were managed surgically in the OR. On the other hand, seven (77.8%) were managed conservatively. The most important causes that can lead to post-tonsillectomy hemorrhage from the parents' opinion reported as follows: post-surgical inflammation/infection (272, 34.0%), failure to provide healthcare after the operation (271, 33.9%), and failure to provide healthcare and comprehensive preoperative examinations (257, 32.1%).

**Table 4:** Post-surgical complications and bleeding The data were represented as (N) and (%) for participants in each question.

Post-surgery data	No	%
Choose from the following post-tonsillectomy complications that your child developed.		
Post-surgical headaches and nausea	444	52.4%
Post-surgical swelling of the roof of the mouth	439	51.8%
Post-surgical infection	414	48.9%
Were there any bleeding?		
Yes	47	5.5%
No	800	94.5%
Onset of bleeding?		
During surgery	12	25.5%
≤ 24 hours after surgery	14	29.8%
> 24 hours after surgery	21	44.7%
If intra-operative or immediate post-operative bleeding (≤ 24 hours after surgery) occurred, how was it managed?		
Surgically in OR	9	34.6%
Conservative	17	65.4%
If late post-operative bleeding (> 24 hours after surgery) occurred, how was it managed?		
Directed to ED	9	42.9%
At home	12	57.1%
If directed to ED, how was it managed?		
Surgically in OR	2	22.2%
Conservative	7	77.8%
In your opinion, what is the most important cause that can lead to post-tonsillectomy bleeding?		
Post-surgical inflammation/infection	272	34.0%
Failure to provide health care and comprehensive pre-operative examination	257	32.1%
Failure to provide health care and attention after operation	271	33.9%

Table 5 presents the factors associated with bleeding history among children undergone tonsillectomy surgery. Bleeding was reported among (7.3%) of cases undergone the surgery in summer versus (3.8%) of winter cases (P=0.026). Additionally, having bleeding disorder (hemophilia) (23.2%), diabetes mellitus (DM) (30.5%), and rheumatic fever (10.3%) was significantly associated with bleeding (P<0.05). Meanwhile, 51.4% of children on anti-inflammatories had bleeding versus (1.3%) who were not on anti-inflammatories (P=0.00001), and 53.8% of children where the treating medical staff did not ask about anticoagulants or aspirin described above before the operation had bleeding (P=0.00001).

**Table 5:** Factors associated with bleeding history among children who underwent tonsillectomy surgery All statistical methods used were two-tailed with an alpha level of 0.05 considering significance (*) if a P value is less than or equal to 0.05.

Factors	Bleeding				p-value
	Yes		No		
	No	%	No	%	
Child gender					0.418
Male	22	4.9%	423	95.1%	
Female	25	6.2%	377	93.8%	
Child age at the time of surgery					0.656
1-5 years	15	6.5%	217	93.5%	
6-10 years	8	3.8%	200	96.2%	
11-15 years	11	5.9%	175	94.1%	
16-18 years	13	5.9%	208	94.1%	
Season at the time of surgery					0.026*
Winter	16	3.8%	406	96.2%	
Summer	31	7.3%	394	92.7%	
Did any of the tests show the presence of anemia?					0.754
Yes	22	5.3%	394	94.7%	
No	25	5.8%	406	94.2%	
Does your child have any of the following comorbidities?					
Bleeding disorders (hemophilia)	22	23.2%	73	76.8%	0.001*
Morbid obesity	25	5.7%	411	94.3%	0.809
DM	29	30.5%	66	69.5%	0.002*
Chronic tonsillitis	27	6.2%	410	93.8%	0.409
Rheumatic fever	19	10.3%	166	89.7%	0.002*
Did the treating medical staff ask about the conditions described above before the operation?					0.01*
Yes	38	4.9%	734	95.1%	
No	9	12.0%	66	88.0%	
Is your child under anti-inflammatories (ibuprofen)?					0.00001*
Yes	37	51.4%	35	48.6%	
No	10	1.3%	765	98.7%	
Did the treating medical staff ask about the treatment described above before the operation?					0.074
Yes	23	7.4%	288	92.6%	
No	24	4.5%	512	95.5%	
Is your child under anticoagulants or aspirin?					0.0087*
Yes	23	8.6%	245	91.4%	
No	24	4.4%	555	95.6%	
Did the treating medical staff ask about the treatment described above before the operation?					0.00001*
Yes	16	2.0%	766	98.0%	
No	35	53.8%	30	46.2%	
Were all recommended directions of the treating medical staff followed before and after the surgery?					0.153
Yes	17	4.3%	375	95.7%	
No	30	6.6%	425	93.4%	

## Discussion

The current study aimed to investigate parents' perceptions of post-tonsillectomy bleeding following their child's tonsillectomy. It explored the experiences of parents regarding post-tonsillectomy hemorrhage, including their understanding of risk factors, recognition of symptoms, and actions taken in response to bleeding incidents. The study also assessed factors with bleeding history among children comorbidities, medications, and communication with healthcare professionals. The study showed no gender differences regarding the frequency of tonsillectomy and that there was nearly uniform distribution regarding the child's age at surgery time and season at surgery. The most reported clinical complaints were abdominal pain, voice changes, skin rash and headache, odynophagia, and sore throat, which are the most common signs and symptoms associated with tonsillitis [10-12].

With regard to pre-operative healthcare, nearly half of the children underwent complete laboratory tests before surgery, and also about half of the parents were informed by the healthcare staff about surgery-related complications, and less than half of them were informed how to deal with the complications if they occur. This indicates a shortage in healthcare staff's role in child assessment and parent education, which is questionable and needs further assessment to identify the reasons behind that shortage. Comprehensive child assessment and parent education will help minimize surgery-associated complications [13]. Yang et al. [13] in their study supported the effectiveness of tonsillectomy education using smartphone text messaging in increasing mothers’ knowledge and reducing children's anxiety and surgery complications. Globally, pediatric otolaryngology facilities are starting to include novel patient/parent education programs prior to tonsillectomy in their perioperative procedures [14]. The effectiveness of incorporating these education programs helps minimize children's and parent's anxiety and make them able to deal with all unexpected complications [14,15]. On the other hand, Jain et al. [16] in their study reported that the most common reason for return to the emergency department among tonsillectomy cases was hemorrhage (4.9%). Patients also returned to the ED for preventable reasons such as dehydration, pain, nausea/vomiting, and fevers. They also revealed that pre-operative tonsillectomy education is feasible but is not associated with fewer ED visits and admissions or fewer ED visits for preventable causes. Additionally, Cooper et al. [17] documented that the best way to save money is to forego pre-operative testing. Institutions and groups may find these statistics useful in developing policies around pre-operative coagulation for kids who have not had a bleeding disorder diagnosis yet.

With regard to post-tonsillectomy complications, the vast majority of the children had minor complications, including headache and nausea, infection, and swelling of the roof of the mouth. The minority of the children that were documented by their parents in this study had bleeding. Most of the bleeding cases developed after 24 hours of the surgery. Infection and the lack of pre-operative and postoperative care were equally responsible for causes that can lead to hemorrhage as reported by the parent's point of view. Conservative management to deal with bleeding was reported in nearly half of the cases. This estimated rate of bleeding was within range of the global reported incidence of post-tonsillectomy hemorrhage, occurring at a rate between 0.28% and 20% [18-22]. This wide range of post-tonsillectomy hemorrhage rates reflects the diversity in the otolaryngologic community on how to properly define significant post-tonsillectomy hemorrhage, but most of the cases had secondary bleeding (> 24 hours after surgery), which was the same findings in this study [20-22]. In Saudi Arabia, Aldrees et al. [23] documented that post-tonsillectomy hemorrhage occurred in only 5.3% of tonsillectomies. The current study showed that post-tonsillectomy bleeding was nearly the same and was higher among children with other comorbidities mainly bleeding disorders and DM, having medications that may affect blood clotting and anti-inflammatories with a lack of medical staff care about received medications to stop before surgery. This was similar to the previously reported study's findings about correlates of post-tonsillectomy bleeding [23-25].

This study stands out as it includes individuals from every region of Saudi Arabia. However, we have faced some limitations associated with this study. First, the distribution of the online survey via social network platforms could have shown a bias towards individuals who did not have access to it. Second, the sample that was collected was not equally distributed across the different regions in Saudi Arabia. As a result, the final results and findings that this study showed may not reflect the entire population.

## Conclusions

The current study revealed that there was a shortage regarding the role of healthcare staff in child presurgical assessment and in parents' education regarding expected complications and how to deal with them. Additionally, their role in medication inquiry and informing parents when to stop before surgery was questionable. This explains the incidence of reported bleeding, mainly secondary bleeding (> 24 hours), and the need for surgical intervention for management.
